# Massive Distance Education: Barriers and Challenges in Shifting to a Complete Online Learning Environment

**DOI:** 10.3389/fpsyg.2022.928717

**Published:** 2022-06-23

**Authors:** Ching-Yi Yeh, Chin-Chung Tsai

**Affiliations:** ^1^Program of Learning Sciences, National Taiwan Normal University, Taipei, Taiwan; ^2^Institute for Research Excellence in Learning Sciences, National Taiwan Normal University, Taipei, Taiwan

**Keywords:** massive distance education, barriers, videoconferencing fatigue, videoconferencing refugee, online learning, fully online learning

## Abstract

The global pandemic has dramatically changed how the world functions and impacted all sectors of society including all educational institutions. Government and educators respond with immediate online teaching and learning for all students. Massive distance education has been drawn into the picture to provide non-stop learning in most countries worldwide. This study focuses on examining different orders of barriers educators have encountered during the Covid-19 pandemic. The barriers to massive online teaching and learning included the first-order barrier (technological or external barrier), the second-order barrier (internal barrier or teachers' and parents' beliefs), the third-order barrier (design thinking barrier), and the 2.5th order barrier (the classroom management barrier). Both teachers and students are suffering from unstable or limited internet connectivity and it directly hinders students' rights in the massive online education. Teachers are facing the need for sudden pedagogical redesign while parents are enduring the burden of providing all kinds of support for their children's online learning at home. Some learners are experiencing videoconferencing fatigue and struggling with overwhelming resources and an excessive amount of technology time. This study also identifies a group of forgotten learners, the videoconferencing refugees, who have limited access to the Internet and lost their learning opportunities. From a global perspective, shifting to massive online education may be possible with all four orders of barriers being overcome.

## Introduction

“Lockdown” and “Coronavirus (Covid-19)” have been two of the most popular searched terms on the Internet since 2020. The year 2020 has been an unusual and extremely difficult time for the entire world due to the major outbreak of the covid-19 pandemic (Spinelli et al., 2020). According to the World Health Organization (WHO), there have been more than 300 million confirmed Covid cases globally prior to January 2022. The critical situation has also impacted the educational settings and brought in many unexpected challenges for educators and learners around the world (Dhawan, 2020; Eradze et al., 2021; Muthuprasad et al., 2021). Modern crises such as global warming, pollution, and pandemic, all of which may lead to education fall off in a high-risk society (Pietrocola et al., 2021). Unexpectedly, this global pandemic has affected our living conditions dramatically and changed how we function every day (Spinelli et al., 2020). The sudden dramatic change leaves no time and space for traditional classroom teaching and learning (Kapasia et al., 2020). School across from K12 to universities around the world such as Australian universities faced a sudden shift to online teaching and learning during the pandemic (Smith and Kaya, 2021). All stakeholders need to keep up with many uncertainties of the pandemic, including policymakers, educators, parents, and learners will now have to quickly respond to teaching, learning, and collaborating online through different online learning platforms and tools (Gonzalez et al., 2020; Junus et al., 2021). During the pandemic, all learning modes are shifted online due to the lockdown of campuses and schools (Dhawan, 2020; Asanov et al., 2021; Muthuprasad et al., 2021). Challenges, barriers, and potential concerns appeared when it comes to shifting all teaching and learning to an online mode. As a result, there is an urgent need for teachers and educators to adopt new ways of teaching (Harsha and Bai, 2020; Junus et al., 2021).

## Massive Distance Education

Distance education across different regions and countries in the world has been utilized to fulfill a small student population's learning need. Students who are unable to attend face-to-face classes can participate in distance education courses (Beldarrain, 2006). Distance education, clearly, should rely on technology-assisted instruction or online education (Beldarrain, 2006). In online education, teachers and individual students interact with each other in online learning environments using the Internet and technology (Beldarrain, 2006), some common technology that supports learning are mobile phones, laptops, tablets, computers, etc. (Singh and Thurman, 2019). Different from distance education which offers small-scale learners experience in the same class at the same time, massive online open courses (MOOCs) intend to offer opportunities and facilitate learning outside of the classroom settings (Kop, 2011), meaning an unlimited number of learners can attend the same course at any time globally (Grover et al., 2013; Joia and Lorenzo, 2021).

Now, as all learners are required to attend classes online worldwide, the status quo becomes a massive distance education setting during the pandemic. Unlike MOOCs that offer certain courses online, thousands of daily routine K-12 classes and higher education courses are pushed online entirely during the pandemic, meaning a remarkable number of learners are currently participating in a massive distance education environment worldwide. With the increasing need for online technology-assisted teaching and learning, teachers and learners encounter some barriers and challenges. Reimers and Schleicher (2020) pointed out the need for redesign in all aspects including the roles of teachers and students, curriculum design, classroom activities, assessment, as well as support for student wellbeing. Furthermore, to maintain non-stop learning in basic education, school districts and universities have considered and included online learning tools such as Zoom or Google Meet (Alameri et al., 2020; Rahiem, 2020; Serhan, 2020; Muthuprasad et al., 2021).

Online learning provides learners and faculty members with another way to maintain education during the pandemic (Olivares et al., 2021). Many instructors in higher education have switched to online teaching and focused on technology integration for students' learning at home in most countries during the pandemic (Alameri et al., 2020). With online learning, the government and school districts hope that students can attend classes without being exposed to any potential viruses and maintain proper social distances (Toquero, 2020; UNESCO, 2020). However, there are some potential difficulties and uncertainties when all teachers and students are forced to learn in the same online mode (Hodges et al., 2020; Kapasia et al., 2020; Zalat et al., 2021). Educators, clearly, encounter barriers and challenges in online teaching and learning settings (Reimers and Schleicher, 2020; Engzell et al., 2021).

## Barriers to Technology Integration in Education

Ertmer (1999) proposed a framework elaborating on first-order barriers and second-order barriers for technology integration in education. The first-order barrier includes some external factors that may constrain classroom technology integration, such as lack of adequate access, time, training, and institutional support. These factors are extrinsic to teachers. Furthermore, the author added the second-order barrier, which is more intrinsic to teachers, includes teachers' beliefs in pedagogy, beliefs in technology integration, and teachers' willingness to change (Tsai and Chai, 2012); these are teachers' personal beliefs that may promote or burden the implementation of technology integration in classrooms. In addition, Tsai and Chai (2012) proposed the teachers' design thinking as the third-order barrier to technology integration. Tsai and Chai (2012) further explained that teachers can use design thinking to redesign lessons and offer creative activities to better facilitate different groups of learners' needs.

## Re-Examine the Barriers to Massive Distance Education

As shown in previous studies, teachers face three orders of barriers in classroom technology implementation. Now, it is time to re-examine the three orders of barriers in massive distance education settings, the first-order barrier—technology integration (extrinsic barrier), the second-order barrier—teachers' personal beliefs (intrinsic barrier), and the third-order barrier—design thinking.

Teachers may run into a variety of difficulties and obstacles as they try to integrate technology into their teaching (Ertmer, 1999). Ertmer (1999) categorized the barriers in two different orders. The first-order barrier in technology integration in classrooms is extrinsic, both preservice and in-service teachers may run into first-order barriers in resources such as available technology, sufficient training, planning time, and relevant administrative support (Ertmer, 1999; Lin et al., 2014). Teachers' technological skills directly impact the effectiveness and quality of online teaching (Danchikov et al., 2021). Teachers are also reporting barriers to the lack of access to the internet and devices in e-learning implementation (Almanthari et al., 2020). Moreover, teachers do not have sufficient experience in a fully online learning environment (Lase et al., 2021).

In modern education, students' technological skills become essential in obtaining the learning resources (Rasheed et al., 2020). Danchikov et al. (2021) pointed out that students' technological skills affect the effectiveness of their online learning. Limited internet access directly impacts the parents who work from home and homeschool children during the pandemic (Alba and Kang, 2020; Stelitano et al., 2020). Lack of proper devices and stable internet connectivity hinder e-learning at home (Almanthari et al., 2020). In addition, parents have the extra burden to ensure students have all the learning materials ready and set up the technology for their children to attend online classes (Iivari et al., 2020). In the current situation, parents are also experiencing the burden and barrier of providing and maintaining proper online learning technology for their children (Abuhammad, 2020; Aliyyah et al., 2020; Garbe et al., 2020). Students' participation in online learning activities is interrupted due to deficient internet connectivity in rural areas and the slow internet connection frustrates the learners while trying to access the learning platforms and materials (Muthuprasad et al., 2021). Students find it challenging to stay connected and learn online from home (Rahiem, 2020). Therefore, immediately intervention strategies should be considered to help strengthen the communication and collaboration between schools and parents to better facilitate children's learning (Aliyyah et al., 2020; Manca and Meluzzi, 2020). The factors mentioned above are categorized as first-order barriers.

The second-order barrier is intrinsic, including factors such as teachers' personal beliefs about technology integration, willingness to change, and teachers' pedagogical beliefs (Ertmer, 1999; Tsai and Chai, 2012). Unlike traditional classrooms without technology implementation, teachers may be dealing with multiple changes in teaching methods, assessment, and management styles (Kerr, 1996). These second-order barriers are more likely to hinder classroom technology integration as they are deeply rooted in teachers' personal beliefs (Dede, 1998; Ertmer, 1999). Extensive research has also been done in addressing teachers' beliefs and conceptions of the technology-enhanced learning environment (Ellis et al., 2006; González, 2009, 2010; Sherman and Howard, 2012; Schweighofer and Ebner, 2015; Svihla et al., 2015; Saxena, 2017; Durff and Carter, 2019).

During the pandemic, parents and teachers become the essential support for students to foster a proper attitude toward online learning (Manca and Meluzzi, 2020). Parents are having a difficult time becoming teachers at home due to their inability to provide effective distance learning support for their children (Lase et al., 2021). As participants, parents' and students' intrinsic beliefs about online learning may also affect the learning outcome. Parents' assistance in the massive online education becomes crucial; however, they may not see the positive learning outcome of online learning. Students are dealing with learning challenges with limited non-verbal cues in an online learning environment (Khalil et al., 2020). It becomes challenging for learners to keep learning and stay connected in a comfortable online learning environment (Muthuprasad et al., 2021). In Scull et al. (2020)'s engagement study of Australian university students, they found that students need more guidance on how to ask the right questions and seek help in an online learning environment. In taking distance education courses, major concerns such as time management, motivation, and language skills should be taken into account (Fidalgo et al., 2020). As a result, teachers and school administration at all education levels should seek possible ways to overcome the second-order barrier to further assist the parents and students. These are the crucial factors in the second-order barrier.

In the past, researchers developed very few platforms for totally online teaching. The learning systems and platforms are easier to implement if they could be integrated with traditional face-to-face instruction before the pandemic. Most teachers did not have rich and adequate experiences of totally online education or teaching. Educators are ill-prepared in transitioning into the online teaching setting (Rahiem, 2020). Traditionally, technology integration instruction is designed as a part of face-to-face teaching. Teachers who have experience in teaching in online settings do not encounter as many obstacles as teachers who have none or minimal online teaching experience. Dhawan (2020) stated that teachers will need to find new ways to provide meaningful learning and engage the students in the online setting. Tsai and Chai (2012) pointed out the third-order barrier—the design of teaching strategies. The authors discussed the possibilities of achieving successful technology integration after the first and second-order barriers are being overcome. As the teachers have sufficient technology with adequate pedagogical beliefs, they may still encounter the third-order barrier, which is the need to redesign learning materials to cater to different learners' needs in a completely online setting.

Design thinking skills in education help promote creativity, collaboration, and problem-solving skills (Caruso, 2011; Scheer et al., 2012; Watson, 2015; Henriksen et al., 2017; Lambert et al., 2021; Nguyen et al., 2021). Design thinking is a powerful process to solve problems collaboratively (Deitte and Omary, 2019; Panke, 2019). Tsai and Chai (2012) indicated that design thinking intends to make changes and solve current issues with a creative mindset. With all being said, teachers may face different challenges when it comes to design thinking in online settings (Vallis and Redmond, 2021). Teachers need design thinking skills to overcome the potential challenges of online teaching (Vallis and Redmond, 2021). Muthuprasad et al. (2021) indicated the key factor of a successful online class is interactivity. In addition, they explained that constant meaningful activities help engage the learners in online classes. In massive online education, teachers must create a collaborative online learning environment to enhance the effectiveness of massive online education. Teachers with design thinking skills act as facilitators to provide students with creative learning experiences and guide students to deal with challenges (Noweski et al., 2012; Lambert et al., 2021). The design thinking skill is the third-order barrier.

Apart from all three orders of barriers, Chen et al. (2022) found the 2.5th order barriers: classroom management for totally online teaching. The authors studied the potential barriers to teachers' use of mobile devices in classrooms. They added that when using the mobiles devices to attend online classes, teachers found it challenging to engage the students and maintain their attention. Students were often distracted by other tablet applications or accidently clicked on the wrong button that led to other matters. This would require the teachers to use a different set of classroom management skills specifically for online teaching. They further explained that as all three barriers are overcome, the barrier of classroom management may still affect teachers' willingness to integrate technology in their classrooms. One of the biggest concerns for K-12 teachers and higher education instructors is that they may not have sufficient educational knowledge for online teaching (Ching et al., 2018). Ghateolbahra and Samimi (2021) indicated teachers need to put in extra effort in dealing with online classroom management to provide meaningful learning. In the totally online teaching environment, classroom management becomes another crucial factor that affects the effectiveness of online teaching and learning. Classroom management in the online education environment leads to a new view in primary schools (Lathifah et al., 2020). Teachers can address the online classroom management challenge by setting up comfortable relationships with the students, paying extra attention to in-class disruption, and having inclusion plans for students with special needs (Baker et al., 2016). Constant monitoring of student practice and effective feedback are also important in an online learning environment (Prilop et al., 2021). The classroom management strategy is the 2.5th order barrier.

## Videoconferencing Fatigue and Videoconferencing Refugees

When it comes to online learning challenges, the term “digital divide” was highly discussed in the United States and in Europe (Van Dijk and Hacker, 2003). Hargittai (2003) stated that the digital divide is a social issue representing the gap between those who have Internet access and those who do not have any. Rogers (2001) also discussed the extent to which individuals become a disadvantaged group of people in society due to the lack of access to the Internet. During the current pandemic, as all students are pushed to a massive distance learning environment, similar to the digital divide issue, it is unfortunate to see a countless number of students are losing their learning opportunities and falling behind the curriculum in massive distance education. Currently, many countries face the challenges of not having reliable Internet connectivity or sufficient digital devices (Pokhrel and Chhetri, 2021). Some students are facing the problem of restricted or no electricity (Lathifah et al., 2020). On top of internet connectivity concerns, the students are struggling with issues such as technical problems, utilizing online learning strategies, social isolation, stress, etc. (Elmer et al., 2020; Babicka-Wirkus et al., 2021).

As all teaching and learning shift online entirely during the pandemic, an increasing number of students are required to join the online learning environment (Serhan, 2020). Now, with campus lockdown, many higher education institutions and teachers use common online video conferencing tools such as Google Meets, Microsoft Team, or Zoom (Almendingen et al., 2021; Jindal et al., 2021). Higher education institutions have moved all learning online and utilized web conferencing tools for course content delivery (Bullock et al., 2021). Serhan (2020) states that the use of video conferencing tools is not new in the education field. At the university level, videoconferencing was used during office hours to answer students' questions and concerns regarding course content (Danchikov et al., 2021). During the pandemic, more research attention has been given to the use of video conferencing tools, more specifically, the use of Zoom in K12 to higher education classes (Serhan, 2020; Singhal, 2020; Joia and Lorenzo, 2021; Wiyono et al., 2021). To facilitate learning at home, some school administrators and teachers provide computers, tablets, and Internet access for students to learn online and get through the transition (Serhan, 2020). For students who have sufficient necessary resources at home, they have stable Internet connectivity, computers, laptops, tablets, and even smartphones. These students are the somewhat fortunate ones who have abundant resources. However, these resources can be overwhelming and sometimes disrupting when it is all combined with the online classroom. With the sudden dramatic growth of screen time and intense online learning schedule, the situation can go out of control and easily lead to videoconferencing fatigue for learners in higher education.

Bailenson (2021) defined “Zoom Fatigue” as an exhausting situation where the use of Zoom video conferencing increased dramatically during the pandemic. Fosslien and Duffy (2020) further explain that the intense focus on verbal conversations makes Zoom users debilitating. In K-12 education, all learners have been learning and completing assignments online during the pandemic (García and Weiss, 2020; Serhan, 2020) and there is no foreseeable end date to the current situation. Dorn et al. (2021) point out that students struggle with multiple difficulties in class scheduling, technical issues, and Zoom fatigue. Notably, students are unprepared and overwhelmed with the given information and they may not have all the necessary technological skills to navigate around the online learning platforms. Bullock et al. (2021) mentioned the overuse of technology could lead to extra stress both mentally and physically as more higher education institutions move to online learning. In addition, learners are experiencing a lack of physical interaction such as body gestures and facial expressions or responses in Zoom classes (Peper et al., 2021).

Many students are facing troubles such as attending and logging on to online classes, uploading assignments, getting used to the functions to share their screens or express their opinions, etc. (Goldstein et al., 2020). Moreover, student engagement and learning assessment are unclear in remote learning during the pandemic (Asanov et al., 2021). This group of learners suffer from the overwhelming online learning environment and are potentially experiencing videoconferencing fatigue. Furthermore, they have to stay online all day without any social interaction with their peers (García and Weiss, 2020). Ferri et al. (2020) identified similar situations as social challenges where the students have no social interaction with their teacher or other students and they also lack support from their parents or caretakers who might be working from home in the same space. There is an urgent need for an appropriate and effective remote teaching and learning plan for both teachers and students.

Educators face the fast change into emergent remote teaching and teachers need to rely on educational tools such as computers/ laptops, the Internet, online platforms, social media, etc. to offer a sufficient online learning environment (Svrcek et al., 2022). Besides the challenges and barriers teachers encounter, the student's access to technology at home also affects their learning quality (Muthuprasad et al., 2021). Other than learners who are receiving overwhelming resources and experiencing videoconferencing fatigue, there is another group of learners who are dealing with limited or no access to necessary technology tools or have insufficient internet connectivity and limited access to educational resources (Asanov et al., 2021; Lai and Widmar, 2021). Manca and Meluzzi (2020) pointed out there is a group of underserved students who do not have any access to the necessary technology to attend online classes. In contrast to learners who are experiencing zoom or videoconferencing fatigue, the authors of this paper would call this group of struggling learners the *videoconferencing refugees*.

Videoconferencing refugees, coined by this paper, are the forgotten ones during the pandemic, both emotionally and academically. Unfortunately, they are not just being left behind; they are being forgotten in the online learning environment. As the pandemic transformed learning, the video conferencing refugees lost their fair chances to learn or participate in any learning activities due to insufficient technology, digital resources, or Internet connectivity. Padilla Rodríguez et al. (2021) pointed out that rural area students are suffering from unstable internet connectivity, limited electricity, and unqualified teacher in the online learning environments. The learners do not have any opportunities to continue learning even if they wanted to.

During the pandemic, students from low socioeconomic families are facing the challenge of not having a stable internet connection, proper technology, or parent support (Rahiem, 2020; Azubuike et al., 2021). Learners deal with different levels of online learning difficulties while trying to learn at home (Padilla Rodríguez et al., 2021). Some school districts are providing limited hotspot services for families in rural areas (Lai and Widmar, 2021). The achievement gap among the disadvantaged students widened during the pandemic (Dorn et al., 2021). Kapasia et al. (2020) state that some students are now dealing with an unfavorable learning environment due to the pandemic. From computer technical skills to understanding the academic content in online settings, they have no support and are left with no other choices to maintain basic education; hence, the feeling of isolation and anxiety increases as the lockdown period extends. Online learning can be overwhelming and can lead to extra potential stress on the students (Shanahan et al., 2020). In Shanahan et al.'s study of 768 participants, their results indicate an increasing level of emotional distress during the pandemic. Teachers are reporting that their students are not logging into the online sessions, especially the lower-income students and there is a clear increase in student absences rate in the United States (Goldstein et al., 2020). Videoconferencing refugees do not have stable internet connectivity and access to adequate learning resources or technology simply lose their fair chance to learn as much as their peers. As mentioned above, there is a remarkable number of students who are now required to learn online worldwide. Other than instructions given by their teachers, the students, and the parents are fighting the battle alone at home.

## Overall Challenges in COVID

There are multiple aspects of challenges all stakeholders need to deal with during and after the pandemic. In massive distance education, teachers in K-12 and higher education are facing totally online teaching and lesson redesigning obstacles. Teachers need to find the appropriate ways of expression through the online manner and the usage of different modalities to enrich teaching or course materials. The teaching materials (readings, videos, exercises, etc.) become an influential factor as students are spending more time reading and reviewing the lesson materials on their own at home (Rapanta et al., 2020). Furthermore, the use of various online platforms or the fear of monopolies of certain platforms may affect the quality of totally online teaching and learning. The different platforms offer different interactions among various parties (Kennedy, 2020). K12 to higher education teachers need to know how to effectively use online tools, systems, or modules developed before in such a massive distance education setting. In addition, educators can use new technologies (such as automatic analysis) to detect students' engagement, boredom, frustration, success, or failure in learning. Another challenge is the course redesign for skill-based or internship courses. Educators will have to find ways to accommodate students' learning in skill-based courses in the massive distance education setting. For the skill-based courses, instructors may incorporate immersive virtual reality (VR) as a possible solution to ensure meaningful learning takes place in massive distance education. VR offers faculty and students a meaningful and reality-informed learning experience (Schott and Marshall, 2021). Khalil et al. (2020) mentioned the use of virtual simulation technologies can facilitate clinical practice for medical students. The group chat application is used in sharing information and collaborating globally during the pandemic in medical education; virtual learning can also benefit the fellow-in-training (FIT) in multiple medical areas with proper planning for its implementation (Almarzooq et al., 2020).

As for the learner challenges in massive distance education settings, they may face the expression challenges in using various tools of verbal, written, or visual representations. They will also need extra guidance to reach fluent communication through different modalities such as text, verbal, facial expression, social media, etc. In massive distance education settings, students' self-regulation in both synchronous and asynchronous modes becomes another issue for teachers and parents. Parents' concerns, opinions, and time management toward massive distance education should not be overlooked. In addition, school districts and parents need to consider the investment in the infrastructure of massive distance education and the technical support at different levels including for learners, teachers, schools, and parents. Overall, the quality of massive distance education and online learning should be reexamined.

## Further Thoughts in Global Perspectives

As more vaccines are given to citizens worldwide, perhaps we will be seeing the end of this Covid pandemic; yet, will life go back to normal or will this be a constant change? It is time to consider possible ways to overcome the challenging and overwhelming online learning environment to avoid and decrease the level of videoconferencing fatigue. In the transition to massive online education, how do we move forward in higher education? Engzell et al. (2021) mentioned the pandemic has brought significant impact and concerns in students' learning. In their study of national data of The Netherlands students (*n* ≈ 350,000), they found that learning from home students make minimal or nearly no learning progress, especially the disadvantaged students. The disadvantaged students are at risk while coping with learning from home. Furthermore, the quality of learning is being impacted without thorough online teaching and learning planning. After the technological and pedagogical barriers are being overcome with proper online classroom management strategies and appropriate design thinking, the quality of massive online education could be raised to a satisfactory level. To build on the pandemic online teaching experience in higher education, we can possibly make extensive use of all the online learning data collected during the pandemic in the massive distance education to provide more comprehensive support for precision education. The collected data may be studied to improve the quality of massive online education for colleges and universities. As we move along, it is important to keep in mind that videoconferencing refugees in all education levels, from K-12 to university levels should not be held back or forgotten. What will online teaching be like in the near future for higher education institutions? What will online learning be like in the near future for all learners? With all being said, more research should be done to provide a full scope of e-pedagogy in response to such a global crisis.

## Author Contributions

All authors listed have made a substantial, direct, and intellectual contribution to the work and approved it for publication.

## Funding

This work was financially supported by the Institute for Research Excellence in Learning Sciences of National Taiwan Normal University (NTNU) from the Featured Areas Research Center Program within the framework of the Higher Education Sprout Project by the Ministry of Education (MOE) in Taiwan and Ministry of Science and Technology, Taiwan (Grant numbers: MOST108-2511-H-003-038-MY3 and MOST 109-2511-H-003-013-MY3).

## Conflict of Interest

The authors declare that the research was conducted in the absence of any commercial or financial relationships that could be construed as a potential conflict of interest.

## Publisher's Note

All claims expressed in this article are solely those of the authors and do not necessarily represent those of their affiliated organizations, or those of the publisher, the editors and the reviewers. Any product that may be evaluated in this article, or claim that may be made by its manufacturer, is not guaranteed or endorsed by the publisher.
